# Integrative analysis of gut microbiota and metabolic pathways reveals key microbial and metabolomic alterations in diabetes

**DOI:** 10.1038/s41598-025-09328-w

**Published:** 2025-08-21

**Authors:** Yasser Morsy, Nesma S. Shafie, GP2324 consortium, Mohamed Mostafa, Karim A. Ali, Osama N. Afife, Saja Zedan, Basma A. Darwish, Hala T. Nour, Mai M. Ahmed, Madonna T. Ghaly, Jana Moussa, Kholoud N. Shabaan, Rowan M. Abdelmaksoud, Mostafa T. Younis, Nellie Abdeljaber, Fatma A. Taha, Mohamed H. Mohamed, Esraa Khaled, Zeineldin Ibrahim, Tassnim Sherif, Marwa Amer

**Affiliations:** 1 Department of Gastroenterology and Hepatology, University Hospital of Zürich, Zürich, Switzerland; 2 Department of Bioinformatics and Genomics, College of Biotechnology, Misr University for Science and Technology (MUST), Giza, Egypt

**Keywords:** Gut microbiota, Diabetes, Microbial diversity, Metabolic pathways, LEfSe analysis, Metabolomics, Computational biology and bioinformatics, Microbiology, Endocrine system and metabolic diseases

## Abstract

Type 2 diabetes mellitus (T2DM) is increasingly recognized as a condition influenced by gut microbiota composition and associated metabolic pathways. This study investigated the differences in gut microbial diversity, composition, and metabolomic profiles between diabetic and control individuals. Using 16 S rRNA gene sequencing and metabolomic analyses, we observed significantly higher microbial diversity and evenness in the diabetic group, with distinct clustering patterns as revealed by Principal Coordinate Analysis (PCoA). Taxonomic profiling demonstrated an increased relative abundance of *Bacteroidaceae* and *Lachnospiraceae* in the diabetic group, while *Streptococcaceae* was more prevalent in the control group. LEfSe analysis identified key microbial taxa such as *Bacteroides*, *Blautia*, and *Lachnospiraceae_FCS020_group* enriched in diabetic individuals, suggesting a role in metabolic dysregulation. Metabolomic pathway enrichment analysis revealed significant differences in pathways related to fatty acid metabolism, glucose homeostasis, bile acid metabolism, and amino acid biosynthesis in diabetic individuals. Enriching fatty acid elongation and β-oxidation pathways, alongside disrupted glucose metabolism, indicate profound metabolic changes associated with diabetes. Bile acid metabolism and branched-chain amino acid (BCAA) pathways were also elevated, linking these metabolites to the observed gut microbiota shifts. These findings suggest that diabetes is associated with significant alterations in the gut microbiome’s composition and function, leading to disruptions in critical metabolic pathways. This study provides insights into potential microbial biomarkers and therapeutic targets for improving metabolic health in diabetic patients.

## Introduction

Diabetes Mellitus (DM) is a chronic metabolic characterized by elevated blood glucose levels (hyperglycemia)^1,2^. It represents a major global health challenge, with a global prevalence of 10.5% in 2021, projected to increase to 12.2% by 2045^2–4^. The condition affects both sexes equally, but it is more common among the elderly and in urban populations^4^. DM is classified into Type 1 Diabetes Mellitus (T1DM) and Type 2 Diabetes Mellitus (T2DM). T1DM is caused by the autoimmune destruction of pancreatic ß-islet cells, while T2DM results from insulin resistance and relative insulin deficiency, primarily in adults^1,2,5^.

Recent advances have highlighted the role of the gut microbiota in diabetes^6–8^. This diverse microbial community in the gastrointestinal tract, primarily comprised of *Bacteroides* and *Firmicutes* is essential for metabolic and immune functions^6,7,9–11^. Alterations in gut microbial balance have been linked to metabolic disorders, including diabetes^6–8^. This has led to global research endeavors, as outlined by Ursell et al. (2012), to decode these symbiotic relationships between the gut microbiota and health outcomes, particularly in diabetic conditions^12,13^. Studies like Gowd et al. (2019) have shown associations between microbial dysbiosis and DM onset^14,15^, emphasizing its role in metabolic health.

The gut microbiome may influence diabetes through multiple mechanisms, including the production of short-chain fatty acids, modulation of immune response, impacting gut barrier integrity, and modulating host genetic expression^8–10,14^. These pathways interact in complex ways to affect glucose metabolism and insulin sensitivity^6,7,14,15^.

In this context, our study investigates gut microbiota differences in diabetic versus non-diabetic individuals using 16 S rRNA sequencing. By comparing stool samples, we aim to identify the microbiota signatures that may contribute to DM pathogenesis. Our findings may support the development of microbiota-targeted therapies for diabetes management.

## Materials and methods

### Study population and sample collection

Twenty participants were recruited from the Susad Kafafi Memorial Hospital in Egypt, including 10 patients with Diabetes Mellitus (DM) and 10 non-diabetic individuals. Ethical approval for collecting and analyzing patient stool samples was obtained under reference number 2024/0047 from the Institutional Review Board at Misr University for Science and Technology (MUST-IRB). All participants provided written and signed informed consent prior to sample collection. The stool samples were promptly frozen and stored at − 80 °C until processing. All methods were performed in accordance with the relevant guidelines and regulations, including those outlined by the Declaration of Helsinki and the ethical standards of the responsible institutional committee.

### DNA extraction

Microbial DNA was extracted from 200 mg of each homogenized fecal sample. The DNA extraction was performed according to the manufacturer’s instructions for the QIAamp Fast DNA Stool Mini Kit (Qiagen, Germany). After extraction, the concentration and quality of the DNA were checked using a NanoDrop spectrophotometer. The concentration and purity of the extracted DNA were measured with a NanoDrop spectrophotometer evaluating both concentration (at 260 nm absorbance) and purity (260/280 nm ratio). Subsequently, all the extracted DNA specimens were stored at − 80 °C until the next step.

### 16 S rRNA gene sequencing

Illumina Nextera barcoded, two-step PCR libraries using 16 S V3–V4 marker region primers pairs; 341 F (5′-CCTAYGGGRBGCASCAG-3′) and 806R (5′-GGACTACNNGGGTATCTAAT-3′) to construct the library, and Illumina MiSeq, v2, 2 × 250 bp was utilized for sequencing at Macrogen (Macrogen, 2020). We analyzed 16 S rRNA using the QIIME2 pipeline^16^. Following data quality checks, denoising with DADA2 was conducted to merge paired reads, generating amplicon sequence variants (ASVs)^17^. An alpha rarefaction module ensured sufficient depth to capture most features. We then calculated alpha and beta diversity using the core-metrics-phylogenetic module. The Shannon Diversity Index was employed for alpha analysis to estimate microbial species richness, and Pielou matrices were used for evenness. To quantify community dissimilarity, we utilized Bray-Curtis and UniFrac distance matrices in beta diversity. Principal coordinate analysis (PCoA) was performed to enhance the visualization of beta diversity. Taxonomy was assigned to ASVs using the q2-feature-classifier classify-sklearn naïve Bayes taxonomy classifier against the pre-trained Naïve Bayes silva-132-99-nb-classifier, which was trained on Silva (release 138) full-length sequences. Biomarkers with statistically significant differences between groups were identified through a linear discriminant analysis effect size (LEfSe) analysis based on LEfSe software with default settings, aiming to identify taxa that are differentially abundant among diabetic patients and healthy controls.

### Functional metabolic pathway prediction

To predict metagenome functions and pathway abundance based on 16 S amplicon sequences, we used the PICRUSt2 algorithm^18^. In short, genus and pathway abundance were prepared upon sample aggregation (DM and Healthy). Correlation was run between genus and pathways within each condition, followed by filtering the significant correlation with a value less than 0.001 and a correlation value more or less than 0.7. Since this might lead to multiple correlations (one genus could correlate with more than one pathway), Sankey plots were generated using (networkD3) package and plotted separately for data visualization and interpretation for each condition.

### Metabolomics analysis

Each stool sample, weighing approximately 100 mg, was placed in a 1.5 mL Eppendorf tube. A reconstitution buffer was prepared by mixing acetonitrile and methanol in a 3:1 ratio (v/v). For the initial extraction, 400 µL of this buffer was added to each sample, followed by vortexing for 2 min and ultrasonication for 10 min. The extract was dried and resuspended in water: methanol: acetonitrile (2:1:1) before LC-MS injection. Chromatographic separation utilizing a Waters XSelect HSS T3 column, and mass detection was done in negative ion mode. Data were analyzed using MS-DIAL version 4.9 with HMDB for annotation,, and feature extraction was performed using MasterView with a signal-to-noise threshold > 10.

### Statistical analysis

Statistical analyses were performed in R (version 4.4.3). A pairwise comparison was conducted to compare the relative abundance of taxa between the two conditions (DM and Healthy). The Wilcoxon test was used, and the* P*-value was corrected for multiple comparisons using the Holm–Bonferroni method. Linear discriminant analysis effect size (LEFSe) and the linear discriminant analysis (LDA) score were obtained. Additionally, a pairwise PERMANOVA of beta diversity was performed to assess differences in overall community composition between the two groups using the “adonis” function in the “vegan” package in R. A* p*-value of less than 0.05 was considered significant. The correlation between the considerable genera and enriched pathways was conducted using the corrr package in R and the Sankey plot. The MetaboAnalystR package version 3.0.3 was used for metabolomics analysis. The normalized intensities by the median for each sample were mean-centered and divided by the standard deviation of each variable of all annotated features. A heatmap visually presented hierarchical clustering using the Euclidean method for distance calculation and Ward’s linkage for clustering. Metabolite set enrichment analysis (MSEA) was carried out against the “main" data set, and a generalized linear model was used during the quantitative enrichment analysis. Spearman correlations between genera were calculated using the psych package, with* p*-values adjusted via the Benjamini–Hochberg method. Correlations with |r| > 0.5 were retained. The number of significant associations per genus was summarized and visualized by phylum. The directionality of correlations (positive vs. negative) was displayed using a donut chart, which is the plot style used, adapted from PMID: 40,027,484^19^.

## Results

### Microbial diversity and compositional shifts in diabetic versus control groups

**Fig. 1:**
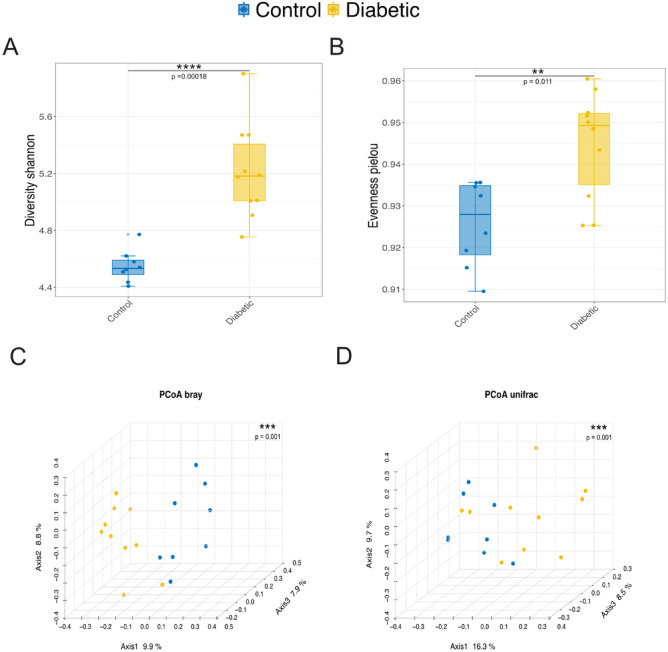
Microbial diversity and compositional differences between control and diabetic groups. (**A**) Boxplot illustrating the Shannon diversity index, reflecting overall microbial richness within the control and diabetic groups. The diabetic group exhibited significantly higher microbial diversity than the control group. (**B**) Boxplot of Pielou’s evenness, which measures the uniformity of species distribution. A higher evenness is observed in the diabetic group, indicating a more homogeneous distribution of microbial taxa relative to the control group. (**C**) Principal Coordinate Analysis (PCoA) plot based on Bray-Curtis dissimilarity, displaying the distinct clustering of microbial communities between control and diabetic groups along Axis 1, which explains 9.9% of the total variation. (**D**) PCoA plot based on unweighted UniFrac distances, further highlighting the separation of microbial communities between control and diabetic samples. Axis 1 accounts for 16.3% of the variation, demonstrating significant differences in phylogenetic composition. Alpha and beta diversity were assessed using the Wilcoxon rank-sum test and PERMANOVA, respectively.

The analysis revealed a higher microbial diversity in the diabetic group compared to the control group, as indicated by the Shannon diversity index (Fig. 1A). Additionally, the diabetic group exhibited a more even distribution of species, with a higher Pielou’s evenness index (Fig. 1B). Conversely, the control group showed a less uniform distribution of species. The Principal Coordinate Analysis (PCoA) based on Bray-Curtis dissimilarity demonstrated distinct separation between the diabetic and control groups, with 9.9% of the total variation (Fig. 1C). Furthermore, the phylogenetic composition of the communities displayed noticeable differences, as evidenced by the PCoA plot based on unweighted UniFrac distances (Fig. 1D). The distinct clustering observed along Axis 1, which accounts for 16.3% of the variation, highlights that the microbial communities in diabetic individuals are compositionally different and phylogenetically distinct from those in the control group. This underscores the impact of diabetes on the gut microbiota’s structure and evolutionary relationships. Furthermore, individuals with diabetes seem to possess a richer and more diverse gut microbiota, suggesting a more balanced microbial ecosystem. These differences reflect significant variations in the relative abundance of microbial species between the two groups.

### Distinct shifts in gut microbial composition and abundance at family and fenus levels in diabetic versus control groups

**Fig. 2:**
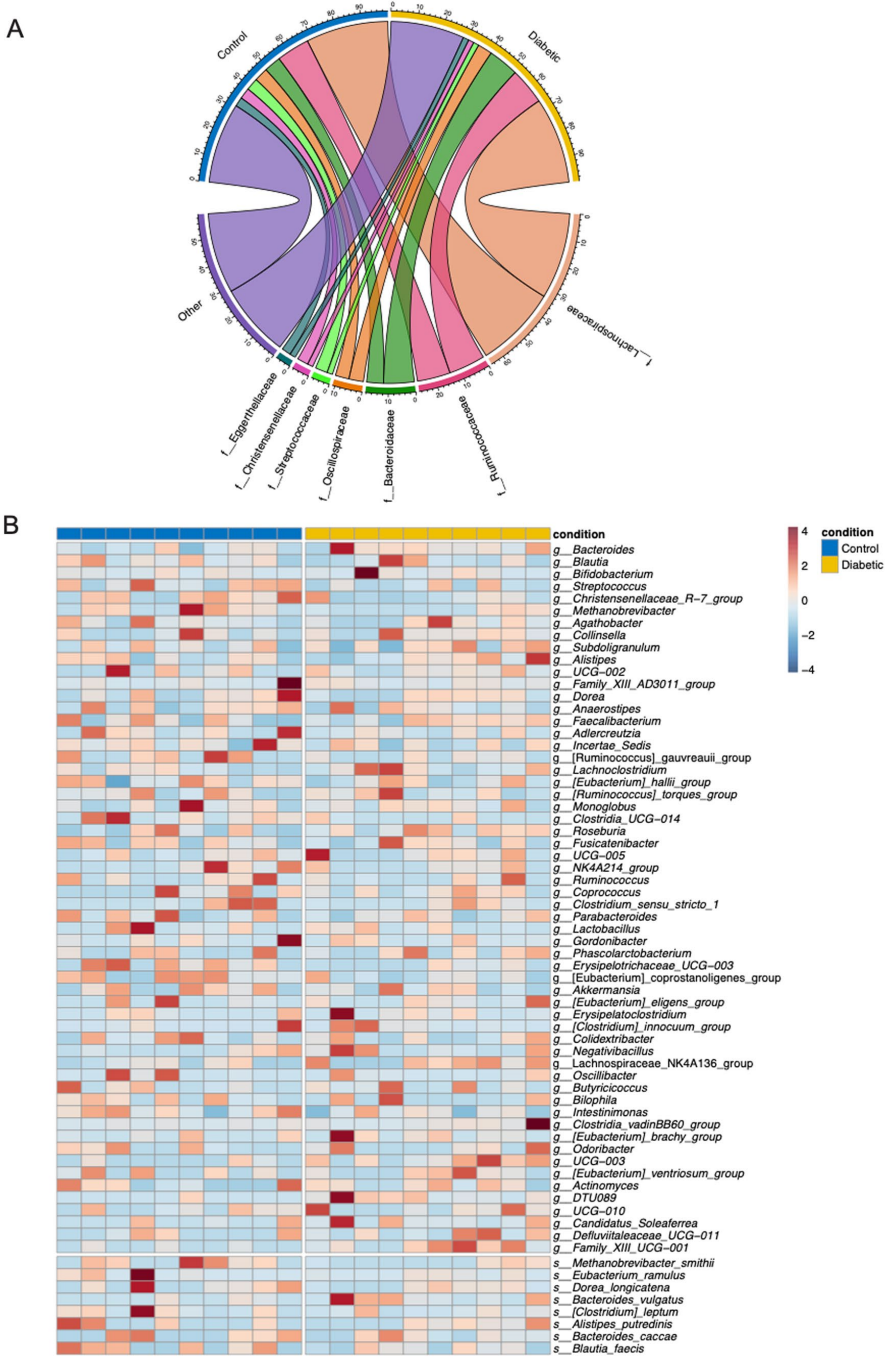
Differential microbial composition and abundance between control and diabetic groups. (**A**) Circos plot illustrating the differential microbial family-level composition between control and diabetic groups. Each segment represents a microbial family, with the connections between the groups showing the relative abundance of each family in the two conditions. Diabetic patients show a higher abundance of families such as Bacteroidaceae and Lachnospiraceae, while families such as *Streptococcaceae* are more prevalent in the control group. (**B**) Heatmap showing the genus-level relative abundance of bacterial taxa across control (blue) and diabetic (yellow) groups. The intensity of the colors represents the relative abundance of each genus, with red indicating higher abundance and blue indicating lower abundance. Notably, genera such as *Bacteroides*, *Blautia*, and *Streptococcus* are more abundant in diabetic patients, whereas *Lachnospiraceae* and *Ruminococcaceae* show higher abundance in the control group. This analysis highlights significant differences assessed by Wilcoxon test and* p*-value were corrected using FDR (*p* < 0.05) in the microbial composition between the two conditions, suggesting a potential role of specific bacterial taxa in the pathophysiology of diabetes.

To explore microbial diversity, a comprehensive taxonomic analysis was performed focusing on differences at both the family and genus taxonomic levels within the microbiome. The study revealed significant differences in the relative abundance of several microbial families between the diabetic and control groups (Fig. 2A). Notably, the diabetic group had a higher relative abundance of *Bacteroidaceae* and *Lachnospiraceae*,* whereas Streptococcaceae* was more abundant in the control group. These findings highlight distinct group-associated microbial patterns rather than temporal changes between the two groups.

Further differences were observed at the genus level (Fig. 2B). The genera *Bacteroides*, *Blautia*, and *Streptococcus* were significantly more abundant in the diabetic group. Conversely, genera linked to *Lachnospiraceae* and *Ruminococcaceae*, such as *Faecalibacterium* and *Roseburia*, were more prevalent in the control group. These distinctions at the genus level further emphasize the divergent microbial profiles between the two conditions. Additionally, other notable genera, such as *Bifidobacterium*, *Methanobrevibacter*, and *Christensenellaceae*, were distributed differently between the two groups. The diabetic group showed a higher relative abundance of *Bifidobacterium*, while *Christensenellaceae* was predominantly found in the control group.

### LEfSe highlights core microbial taxa underpinning gut microbiome divergence in diabetic and control groups

**Fig. 3:**
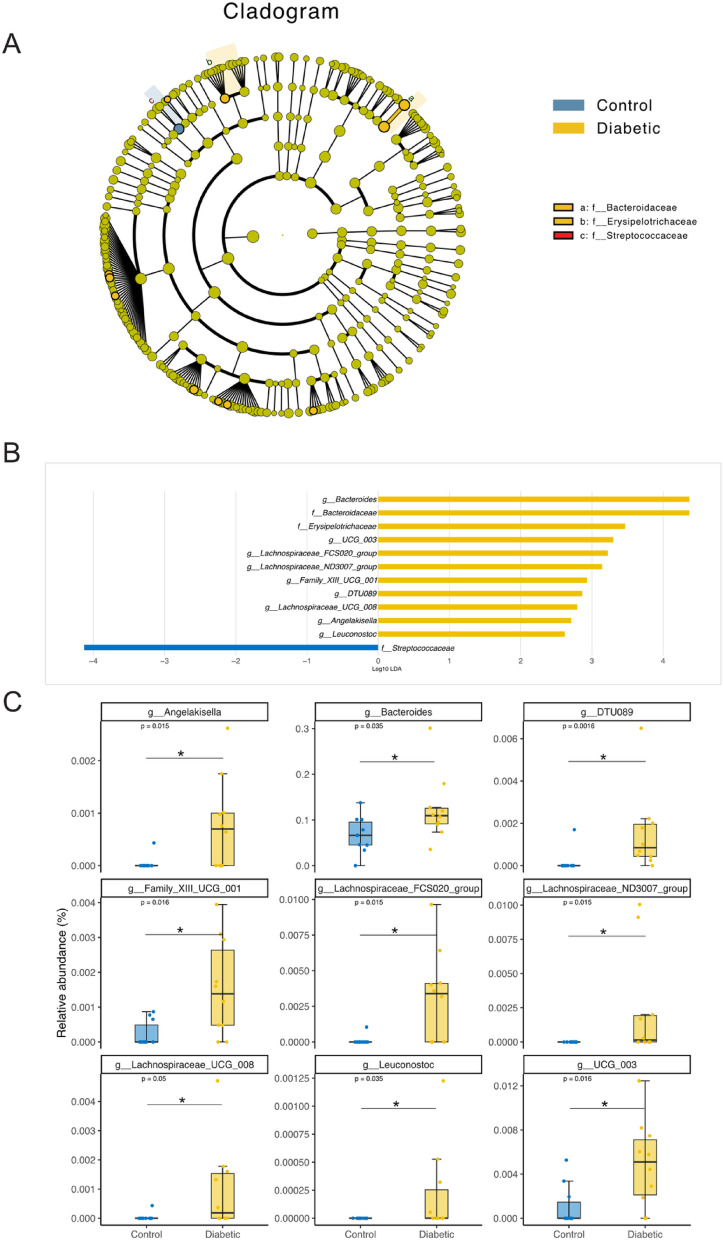
LEfSe analysis of bacterial taxa distinguishing control and diabetic groups.

LEfSe analysis identified significant differences in bacterial taxa between the control and diabetic groups. Taxa from the families *Bacteroidaceae* and *Erysipelotrichaceae* were predominantly enriched in the diabetic group. In contrast, *Streptococcaceae* was more abundant in the control group, as illustrated by the phylogenetic cladogram (Fig. 3A). Supporting these observations, the LDA scores further delineated the microbial differences between the groups. Taxa with positive LDA scores, including *Bacteroides* and members of the *Lachnospiraceae* family, were predominantly associated with the diabetic group. Conversely, negative LDA scores pointed to taxa like *Streptococcus* and *Leuconostoc*, which were more abundant in the control group (Fig. 3B). Additionally, boxplots displaying the relative abundance of key bacterial genera provided a clear illustration of the divergences between the groups (Fig. 3C). Genera such as *Bacteroides*, *Lachnospiraceae*_*FCS020*_*group*, and *Angelakisella* were significantly enriched in the diabetic group, suggesting their potential involvement in disease-related microbial shifts. On the other hand, the genera *Leuconostoc* and *Streptococcus* were more prevalent in the control group, reinforcing the distinct microbial profiles of healthy individuals. The statistical significance of these findings further highlights the robustness of the observed differences in bacterial communities between the diabetic and control groups.

(**A**) Cladogram showing the phylogenetic distribution of bacterial taxa enriched in control (blue) and diabetic (yellow) groups based on Linear Discriminant Analysis Effect Size (LEfSe). Taxa enriched in the diabetic group are primarily from the families *Bacteroidaceae* and *Erysipelotrichaceae*, while *Streptococcaceae* is more abundant in the control group. (**B**) Linear Discriminant Analysis (LDA) scores highlighting bacterial taxa with significant differential abundance between the control and diabetic groups. Positive LDA scores (yellow bars) represent taxa enriched in the diabetic group, while negative LDA scores (blue bars) represent taxa enriched in the control group. (**C**) Boxplots of the relative abundance of key bacterial genera identified by LEfSe analysis. Genera such as *Bacteroides*, *Lachnospiraceae_FCS020_group*, and *Angelakisella* are significantly more abundant in diabetic patients, while *Leuconostoc* and *Streptococcus* are more prevalent in the control group. Statistical significance is indicated with adjusted* p*-values (*p* < 0.05) using Wilcoxon test for each comparison.

### Distinct microbial pathways and taxa associations characterize metabolic alterations in diabetic gut microbiomes

**Fig. 4:**
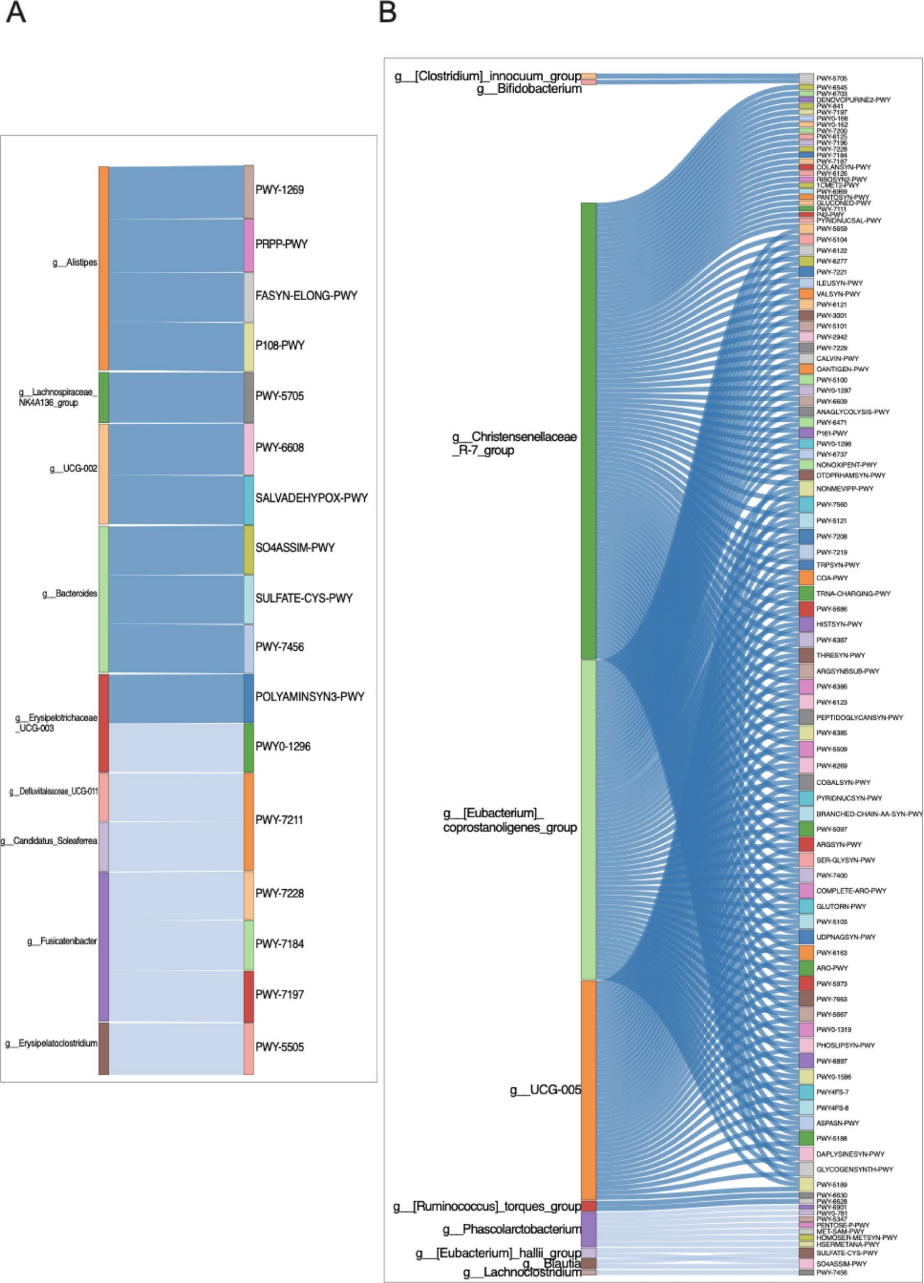
Functional pathway predictions and microbial taxa associations in control and diabetic groups. (**A**) Control group. (**B**) Diabetic group. Each Sankey plot illustrates significant correlations (adjusted *p* < 0.05) between differentially abundant microbial genera and functionally enriched metabolic pathways. Correlations were calculated using Spearman’s method, with a threshold of |r| ≥ 0.7. Dark blue ribbons indicate strong positive correlations (*r* > 0.7), while light blue ribbons represent strong negative correlations (*r* < − 0.7). Only taxa and pathways with both statistical significance and functional relevance are displayed, highlighting distinct microbe–function associations in each group.

Of the 375 predicted metabolic pathways, 233 showed significant differences between the control and diabetic groups. To explore the association of these pathways with specific bacterial genera altered in the diabetic group, participants were initially divided into control and diabetic groups. A correlation analysis was subsequently conducted. A strict threshold was implemented to ensure robust and meaningful associations: only pathways with a* p*-value below 0.0001 were included in the final correlation analysis. This process resulted in 159 pathways correlated with 59 altered bacterial genera. The analysis was further refined by applying a correlation cutoff of 0.7, along with a* p*-value threshold of less than 0.005. This aided in identifying the strongest associations between metabolic pathways and bacterial genera. These results demonstrated the differences between bacterial genera and metabolic pathways under the two conditions. While *Bacteroides* and *Alistipes* were positively correlated with seven different pathways in the control group, *Fusicatenibacter* was negatively correlated with three distinct pathways (Fig. 4A). Conversely, in the diabetic group, we found that *Christensenellaceae*, *Eubacterium coprostanoligenes* group, and UCG-005 positively contributed to most of the altered pathways (Fig. 4B).

### Metabolomic profiles in diabetic versus control groups

**Fig. 5:**
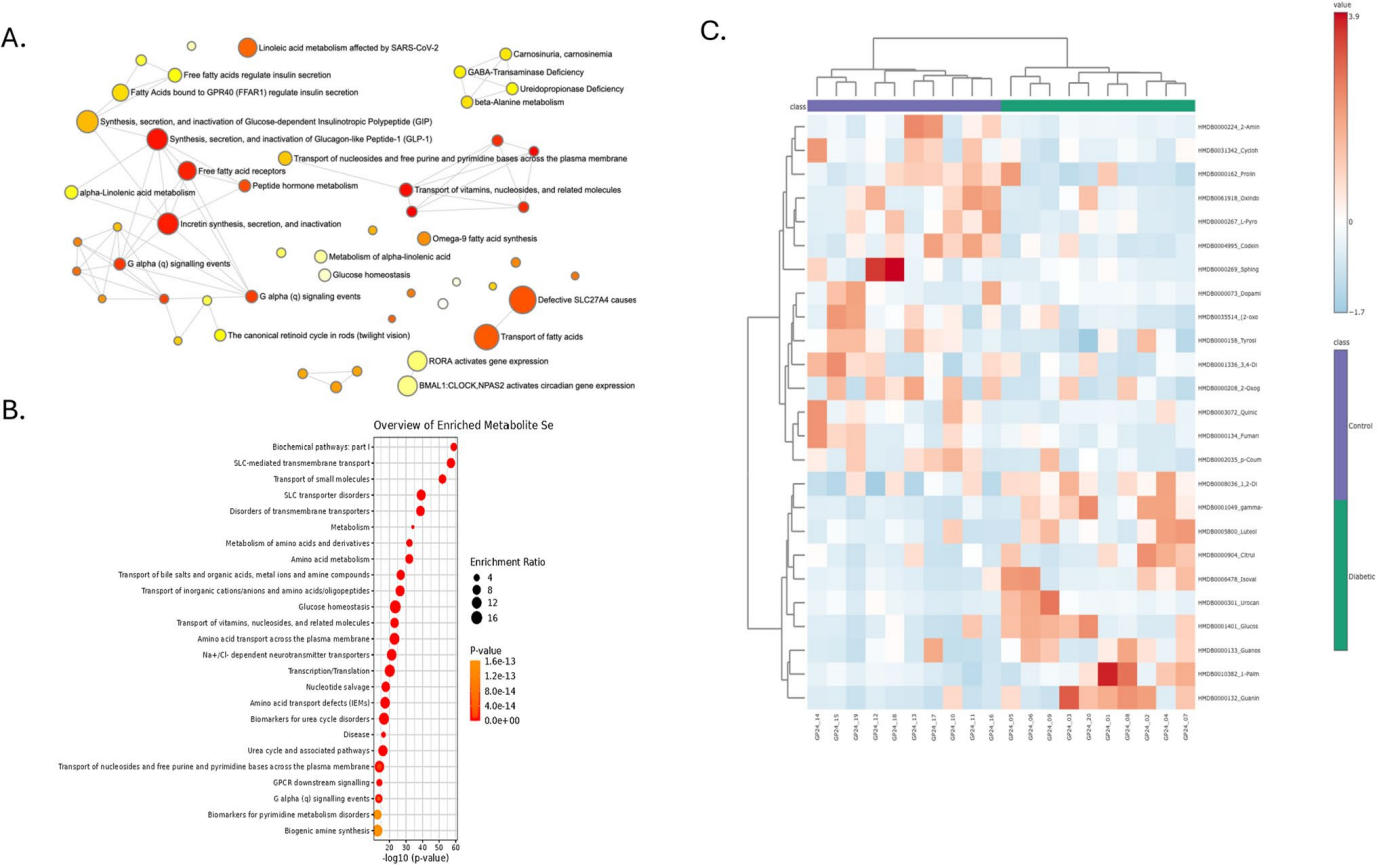
Metabolite Pathway Enrichment and Abundance Differences between Diabetic and Control Groups. (**A**) Network plot illustrating enriched metabolic pathways in diabetic versus control groups. Nodes represent pathways, with node size corresponding to the enrichment ratio and node color indicating significance (*p*-value). Pathways related to fatty acid metabolism, glucose homeostasis, and amino acid biosynthesis significantly enrich the diabetic group. (**B**) Bar plot showing the top enriched metabolic pathways based on enrichment ratios and* p*-values. Pathways such as bile acid metabolism, glucose homeostasis, and amino acid transport are highly enriched in diabetic samples. (**C**) Heatmap representing the relative abundance of key metabolites across control and diabetic groups. Red indicates higher abundance, and blue represents lower abundance. Clustering shows distinct metabolite profiles between control and diabetic individuals, highlighting potential biomarkers and metabolic shifts associated with diabetes.

Metabolomic analysis revealed differences in key metabolic pathways between diabetic and control groups. Pathway enrichment analysis identified fatty acid metabolism, glucose regulation, bile acid metabolism, and amino acid biosynthesis as significantly enriched in the diabetic group (Fig. 5A). Pathways related to fatty acid metabolism, particularly fatty acid elongation and β-oxidation, were more pronounced in diabetic individuals. These pathways displayed a high enrichment ratio, indicating changes in lipid metabolism in the diabetic group compared to controls (Fig. 5B). Additionally, pathways related to glucose homeostasis, such as insulin signaling and hexose metabolism, were enriched in the diabetic group, reflecting differences in glucose metabolic processes. Bile acid metabolism pathways were notably enriched in the diabetic group. Specifically, primary bile acid synthesis showed a significant enrichment ratio, pointing to potential changes in bile acid processing in diabetic individuals. Amino acid biosynthesis and transport pathways, including those for branched-chain amino acids (BCAAs) and aromatic amino acids, were also significantly enriched, suggesting alterations in amino acid metabolism in the diabetic group. The heatmap of metabolite abundances (Fig. 5C) revealed distinct clustering between the diabetic and control groups. Diabetic individuals displayed higher metabolites associated with the enriched pathways, including metabolites linked to fatty acid metabolism, glucose regulation, and bile acid synthesis. This clustering pattern supports the significant differences in metabolite profiles between the groups.

### Co-abundance correlation analysis reveals restructured microbial interactions in diabetes

**Fig. 6:**
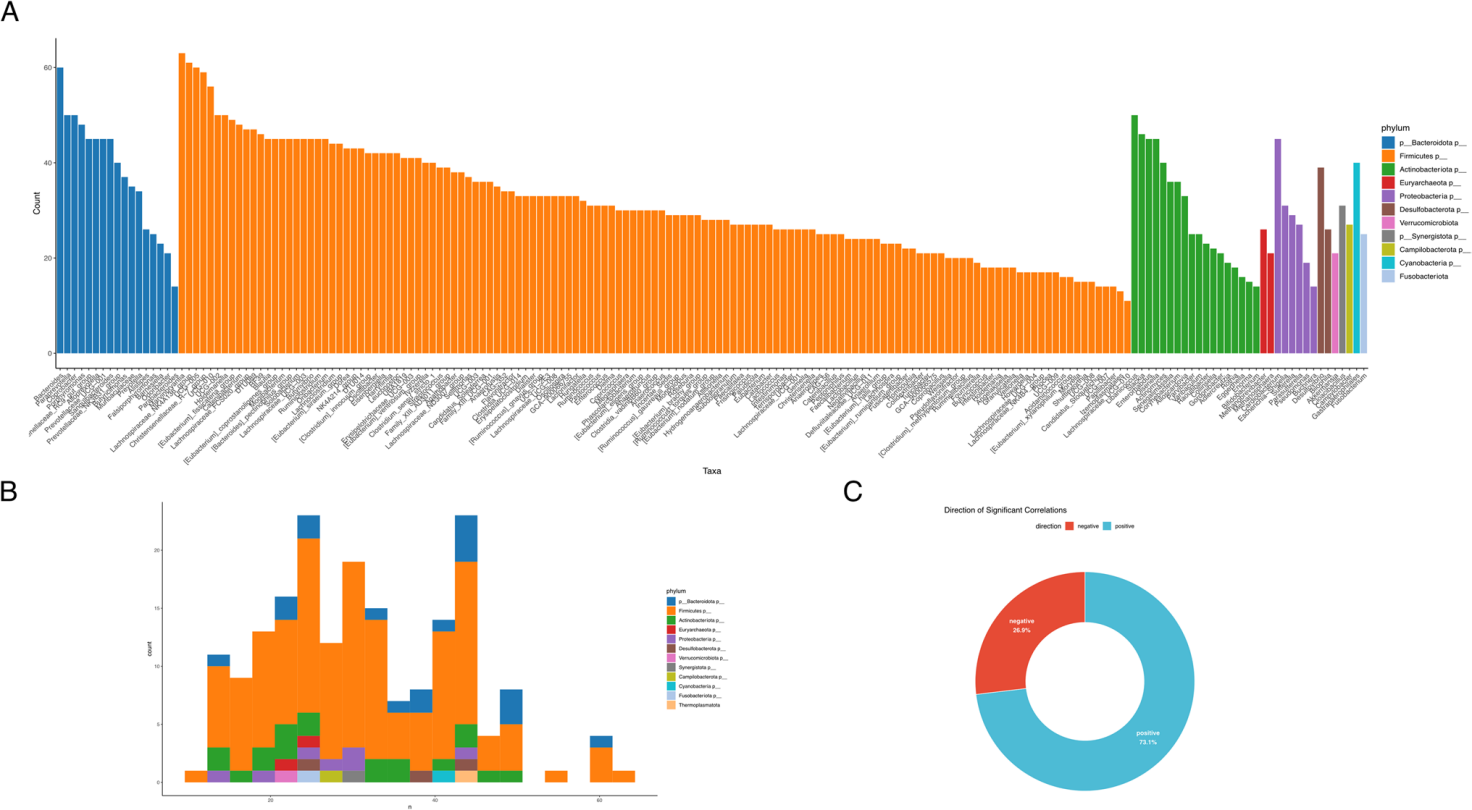
Co-abundance correlation analysis of microbial taxa in diabetic subjects. (**A**) Bar plot showing the number of statistically significant pairwise correlations per taxon, colored by phylum. The height of each bar represents the number of significant associations that the taxon forms with others (Spearman correlation, FDR-adjusted *p* < 0.05). (**B**) Histogram depicting the distribution of taxa by degree (number of connections), stratified by *phylum*. (**C**) Donut chart illustrating the directionality of significant correlations. Positive correlations (blue) accounted for 73.1%, while negative correlations (red) represented 26.9%, indicating that most microbial interactions in the diabetic group were co-occurring rather than mutually exclusive.

To elucidate potential alterations in microbial ecological structure associated with type 2 diabetes, we performed a co-abundance correlation analysis based on Spearman’s rank correlations with FDR-adjusted *p*-values. This analysis revealed a substantial shift in microbial interaction dynamics, with the most significant correlations being positive (73.1%) and a smaller fraction negative (26.9%) (Fig. 6C). The predominance of positive correlations suggests enhanced co-occurrence and potential cooperative behavior among microbial taxa in the diabetic gut environment. Taxa belonging to the phyla *Firmicutes* and *Bacteroidota* were among the most interconnected, exhibiting the highest number of statistically significant associations (Fig. 6A). Furthermore, degree distribution analysis indicated that several taxa serve as highly connected nodes, or potential hubs, within the microbial network (Fig. 6B), implying their key role in structuring microbial communities in diabetes. These findings indicate that diabetes is not only characterized by taxonomic and functional shifts but also by a reorganization of microbial interaction networks - hallmarks of ecological dysbiosis that may influence host metabolic outcomes.

## Discussion

The study investigated the gut microbiota composition and metabolomic profiles of stool samples collected from 10 individuals diagnosed with Diabetes Mellitus (DM) and 10 non-diabetic controls in Egypt. Significant differences in microbial and metabolic characteristics between the two groups were identified, supporting the increasing evidence that changes in gut microbiota are linked to metabolic disorders, such as DM. The notable variations in microbial diversity, composition, and metabolite profiles between the diabetic and control groups emphasize the intricate relationship between gut microbiota and host metabolism, particularly in the context of diabetes. The microbial diversity analysis showed a significantly higher Shannon diversity index and evenness in the diabetic group, indicating a more diverse and evenly distributed microbial community. Our finding of increased Shannon diversity and evenness in the diabetic group contrasts with some Western studies but is consistent with reports from Egypt, Saudi Arabia, and the UAE, where higher alpha diversity has also been observed in diabetic individuals^20–23^. This variation likely reflects population-specific factors such as diet, medication use, and baseline microbiota composition. Moreover, a recent meta-analysis reported no consistent trend in alpha diversity across diabetes studies, highlighting methodological and regional heterogeneity^24^. These results suggest that elevated diversity in diabetes may reflect dysbiosis rather than microbial health.

The observed clustering in the Principal Coordinate Analysis (PCoA) plots, regarding both Bray-Curtis dissimilarity and unweighted UniFrac distances, highlights significant differences in the overall composition and phylogenetic structure of the gut microbiota between diabetic and control individuals Taxonomic profiling further supports this compositional shift, which showed the enrichment of families such as *Bacteroidaceae* and *Lachnospiraceae* in the diabetic group and *Streptococcaceae* in the control group. This finding aligns with previous studies that link metabolic disorders, such as diabetes, to shifts in gut microbial diversity^25,26^. Conversely, we observed an enrichment of *Intestinibacter* and *Lachnospiraceae* NK4A136 group in the gut microbiota of DM patients. *Intestinibacter* has been previously linked to metabolic dysfunction and inflammation, with some studies suggesting its association with reduced SCFA production and increased gut permeability, both of which are implicated in diabetes progression^27^. These results indicate that diabetes is associated with profound changes in gut microbial ecosystems, particularly in taxa that are known to influence metabolic health and inflammation.

LEfSe analysis revealed specific microbial taxa with differential abundance between two groups. Increased levels of *Bacteroides* and *Lachnospiraceae* in diabetic individuals are consistent with findings that link these taxa to inflammation and metabolic dysregulation^26,28^. In contrast, the higher presence of *Streptococcus* in the control group may indicate a protective role in gut homeostasis. These microbial signatures offer insights into how gut microbiota may affect metabolic processes in diabetes.

The functional implications of these microbial shifts were further explored through the predicted functional pathway analysis and metabolite profiling. Pathway enrichment analysis highlighted several critical metabolic pathways significantly enriched in diabetic individuals, including those involved in fatty acid metabolism, glucose homeostasis, bile acid metabolism, and amino acid biosynthesis. These findings suggest that alterations in host metabolic processes accompany changes in the gut microbiota. For instance, the enrichment of fatty acid elongation and β-oxidation pathways in diabetic individuals indicates disrupted lipid metabolism, a hallmark of metabolic disorders such as type 2 diabetes^29^. Similarly, the enrichment of glucose metabolism pathways in the diabetic group reflects the impaired glucose regulation associated with diabetes.

Bile acid metabolism represents another critical pathway that is significantly enriched in the diabetic group. Bile acids play a vital role in lipid digestion and glucose metabolism, and their dysregulation has been associated with metabolic diseases^30^. The increased presence of bile acid-related pathways in diabetic individuals may contribute to the observed metabolic disturbances. Furthermore, the enrichment of amino acid biosynthesis and transport pathways, particularly for branched-chain amino acids (BCAAs), offers additional evidence of altered nutrient metabolism in diabetes. Elevated BCAA levels have been linked to insulin resistance and metabolic dysfunction^31^, indicating that these pathways may influence the metabolic phenotype of diabetes.

Changes in stool metabolomic profiles were observed alongside these microbial alterations. The metabolite abundance heatmap reveals further metabolic differences between the diabetic and control groups. Individuals with diabetes exhibited higher levels of metabolites associated with enriched pathways, such as fatty acid derivatives, glucose-related intermediates, and bile acids. These metabolites strengthen the idea that diabetes involves significant metabolic changes, especially in lipid and glucose metabolism. The clustering of these metabolites in diabetic individuals indicates that distinct metabolite signatures may serve as potential biomarkers for diabetes and its progression.

We conducted a co-abundance correlation analysis to further explore microbial dysbiosis, which revealed a predominance of positive associations among taxa in the diabetic group. This shift suggests altered microbial interactions and increased co-occurrence, particularly among *Firmicutes* and *Bacteroidota*. These findings highlight a reorganization of microbial networks in diabetes, reflecting ecological dysbiosis that may influence metabolic stability and host-microbiota interactions.

Our study offers promising insights into the link between gut microbiota and diabetes, though it does have limitations. The small sample size of 10 individuals per group may limit generalizability, highlighting the necessity for larger, more diverse cohorts to validate our findings and capture individual variability. Importantly, our metabolomic analysis underscores the functional impacts of changes in gut microbiota. Further research using integrated multi-omics approaches is crucial to elucidate the mechanistic connections between microbial taxa and metabolic pathways. Tackling these aspects can enhance understanding and contribute to developing microbiome-based therapies to restore metabolic balance in diabetic patients.

This study’s combined microbiome and metabolomic analyses illustrate a complex interplay between gut microbial communities and host metabolism in diabetes. Changes in microbial composition, alongside modifications in essential metabolic pathways, underscore the possible influence of gut microbiota on the metabolic framework of diabetes. These results lay the groundwork for future investigations aimed at creating microbiome-targeted treatments to restore metabolic balance in individuals with diabetes.

## Data Availability

Sequence data that support the findings of this study have been deposited in the NCBI bioproject database with the primary accession code PRJNA1240603.
